# Comparison of multidirectional jump performance and lower limb passive range of motion profile between soccer and basketball young players

**DOI:** 10.1371/journal.pone.0245277

**Published:** 2021-01-07

**Authors:** Marta Domínguez-Díez, Daniel Castillo, Javier Raya-González, Silvia Sánchez-Díaz, María Soto-Célix, Tara Rendo-Urteaga, Ángel Lago-Rodríguez

**Affiliations:** Faculty of Health Sciences, Universidad Isabel I, Burgos, Spain; Instituto Politecnico de Viana do Castelo, PORTUGAL

## Abstract

This study was performed aimed at comparing multidirectional bilateral and unilateral jump performance and passive range of motion (ROM) of lower limbs between soccer and basketball young players and evaluating associations between inter-limb ROM asymmetry and bilateral jump performance. A total of 67 young male athletes participated in this study, who were classified as soccer (n = 40; 15.55 ± 1.5 y; 1.76 ± 0.12 m; 58.15 ± 10.82 kg; 19.84 ± 2.98 kg·m^2^) and basketball (n = 27; 15.7 ± 1.66 y; 1.76 ± 0.12 m; 62.33 ± 16.57 kg; 19.84 ± 2.98 kg·m^2^) players. Participants were asked to perform bilateral and unilateral multidirectional jumps, and passive ROM of hip (flexion, extension and abduction), knee (flexion) and ankle (dorsiflexion) joints was also assessed. Significant between-group differences were observed for hip extension with flexed knee ROM in dominant (soccer: 142.43 ± 7.74°; basketball: 148.63 ± 8.10°) and non-dominant (soccer: 144.38 ± 8.36°; basketball: 148.63 ± 6.45°) legs; hip flexion with flexed knee ROM in dominant (soccer: 13.26 ± 4.71°; basketball: 9.96 ± 3.42°) and non-dominant (soccer: 12.86 ± 4.55°; basketball: 9.70 ± 3.62°) legs; and for the ratio of hip abduction (soccer: 1.02 ± 0.08; basketball: 0.97 ± 0.11). However, no significant between-group differences were observed for bilateral and unilateral jump capacity, or for inter-limb asymmetries (dominant vs. non-dominant leg). Finally, no associations were observed between ROM ratio (dominant vs. non-dominant leg) and bilateral jump performance. These findings lead to the suggestion that differences on passive ROM values in young male athletes may be sport-specific. Additionally, there seems to be need for the implementation of training strategies specifically aimed at improving bilateral or unilateral jump ability, or at diminishing inter limb passive ROM differences in order to improve multidirectional jump performance for neither soccer nor basketball youth male players.

## Introduction

Team sports athletes need to repeatedly perform high-intensity actions, such as sprinting, change of direction, and jumping [1, 2], thus requiring similar neuromuscular adaptations to develop performance in sports: increased speed, strength and power [3, 4]. Repetitive performance of sprinting, change of direction, and jumping, during training and competition, requires the implementation of intense accelerations and decelerations, besides peaks of impact forces, which are directly associated with decreased neuromuscular performance and joint overload, both factors leading to higher injury risk [1, 5]. However, due to sport-specific playing field size, rules and tactical demands, considerable variability exists across sports and ages for straight-line running, lateral movement, cutting, and jumping [6].

Basketball and soccer are among the most popular team sports worldwide. Whereas soccer is practiced by approximately 265 million people worldwide [7] around 450 million people practice basketball [8]. As for the sport-specific physical demands of these two team sport modalities, soccer demands greater number of movements performed in the sagittal plane (i.e. sprinting) [9], whereas basketball players perform more movements in the frontal plane (i.e. shuffling), and require greater jumping demands [6, 10]. Thus, a rigorous training approach focused on sport-specific actions is needed across all age categories in order to achieve the highest on-field competence, while minimizing the risk of injury [11]. In this regard, comparing neuromuscular performance from young athletes involved in soccer and basketball would allow coaches to improve training programs for these team sports modalities [1].

Jumping ability is crucial for successful performance of several team sports’ fundamental motor skills, in addition to being the most widely used task to improve and indirectly measure lower limb power in multidirectional sports [12, 13]. Although some authors have suggested that jumping ability development may be influenced by sport-specific training adaptations in team sports young athletes [14], evidence has shown no differences between soccer and basketball players for vertical bilateral jump performance, suggesting no specific training adaptation required for these athletes [15, 16]. Nevertheless, most jumps and propulsion forces in these sports are generated unilaterally, requiring the development of multidirectional power to favour sport-specific skills and muscle actions performed during training and competition. Whereas unilateral vertical power would contribute to speed generation, unilateral horizontal and lateral power would determine accelerations generated after a 180° cut, and transitions to lateral shuffling [17]. Thus, it is necessary to assess unilateral multiplanar jumping ability to reproduce sport-specific movement patterns and power characteristics of team sports [17–19]. Furthermore, considering there is growing interest in evaluating the effects of inter-limb asymmetries on jumping performance, a sport-specific multidirectional unilateral jump approach is likely to help improve the strength and conditioning of team sports athletes [17, 20, 21]. In this regard, Gonzalo-Skok et al. [22] showed that unilateral strength training interventions led to greater enhancements of actions mostly relying on the application of unilateral forces, besides resulting in a diminished risk of injury by means of reducing between-limb performance differences (i.e. asymmetry) in young basketball male players, when compared to a bilateral strength training intervention. In addition, Madruga-Parera et al. [23] suggested that multi-directional jumping asymmetries are detrimental to jumping, change of direction and repeated sprint performance in young handball players.

Flexibility is also considered a key component for health and sport performance and is defined by The American College of Sports Medicine [24] as the capacity of a joint to move through its entire range of motion (ROM) [25]. In this regard, it has been shown that ROM deficits are associated with impaired technical skills and sport performance [26–29], and may lead to higher risk of muscle injury [29–31]. Thus, several attempts have been made trying to evaluate factors determining flexibility in young and adult team sport athletes, showing that dominant laterality [32, 33], age [34–36], competitive level [37], tactical position [38] and sex [39], are among such factors. Additionally, it has been suggested that ROM thresholds should be both sport- [40] and age-specific [41], due to adaptions that may result from repetitive performance of the same actions (i.e. straight-line running, lateral movement, cutting and jumping), and several attempts have been made to try to define sport-specific ROM values. Therefore, ROM differences would be expected for athletes from sport modalities where different actions are performed (i.e. soccer vs basketball). In this regard, previous studies have evaluated ROM values from lower limbs in young population, reporting controversial results. Whereas Onate et al. [42] observed differences for active ROM measures across ages and sport modalities, Hogg et al. [43], did not find differences across sports but across genres, with young females showing greater passive ROM than males. Thus, further work is needed in order to better define a lower limb ROM profile between sports in young athletes.

The assessment of inter-limb asymmetries allows the comparison of the performance of one limb with the contralateral limb (i.e. dominant vs non-dominant, stronger vs weaker, injured vs non-injured). Studies have primarily focused on evaluating the associations between inter-limb asymmetries and injury risk [44], although associations of inter-limb asymmetries with physical and sport performance have also been assessed [21]. Evidence suggests that athletes with inter-limb asymmetries > 10% are at higher risk of anterior cruciate ligament injury [45], and are prone to show impaired physical performance [46, 47]. However, there is still controversy regarding the existence of a clear relation between inter-limb asymmetry and physical and sport performance, and especially regarding a potential relationship between inter-limb asymmetries and jumping tasks [21]. Given that greater asymmetry values would be expected for sport modalities in which actions are performed with a clear limb dominance [20] such as soccer and basketball, it would be interesting to evaluate the association between inter-limb ROM asymmetries and bilateral multidirectional jump performance in young soccer and basketball players, since it may allow coaches to adjust training to sport-specific demands.

Therefore, the aim of this study was threefold: i) to compare bilateral and unilateral jumping capacity between youth soccer and basketball players, along with inter-limb jumping asymmetries; ii) to compare ROM of lower limbs between youth soccer and basketball players, and ROM ratio between dominant and non-dominant leg; and iii) to explore associations between bilateral jump performance and ROM ratio for each sport modality.

## Materials and methods

### Experimental design

This study used a descriptive and correlational design to examine differences in jumping performance and passive ROM between soccer and basketball players, and to determine potential associations between inter-limb asymmetries based on measures of jumping performance and passive ROM. All measurements were executed in a single session, performed in the morning at an indoor private room under standard environmental conditions, with participants wearing the gear normally used during training. All testing was supervised by a group of four researchers accredited in strength and conditioning and ROM. Participants performed a standardized warm-up followed, in counterbalanced order, by jumping performance tests and measures of passive ROM of lower limbs. Jumping tests included countermovement jumps and standing broad jumps, performed both bilaterally and unilaterally, in addition to lateral jumps performed unilaterally. In order to avoid any learning effects for jumping measures, two familiarization sessions took place during participants’ physical training sessions performed during the two weeks preceding testing. Participants were asked to refrain from strenuous exercise 48 hours before each testing session and to adhere to their usual diet.

### Participants

A total of 67 young male athletes participated in this study. Forty belonged to a soccer club academy (age: 15.55 ± 1.5 years. height: 1.67 ± 0.12. body mass: 58.15 ± 10.82 kg. body mass index: 20.77 ± 2.49 kg·m^2^) and 27 belonged to a basketball academy (age: 15.7 ± 1.66 years. height: 1.76 ± 0.12 cm. body mass: 62.33 ± 16.57 kg. body mass index: 19.84 ± 2.98 kg·m^2^). All participants trained three times per week (approximately 75 min per session) and competed weekly. Inclusion criteria required that participants completed all the tests, whereas participants were excluded if they had experienced an injury in the preceding 3 months. All participants and their respective parents or guardians were informed about the experimental procedures, along with potential risks and benefits associated with participation in the study. They then signed informed assent and consent forms, respectively. The study was conducted according to the Declaration of Helsinki (2013), and approval was granted by the research ethics committee of University Isabel I (Code: FUi1-PI002).

### Procedures

Participants performed jump and ROM tests in a counterbalanced order, which were preceded by a standardized warm-up consisting of 10 min self-paced low-intensity running and 10 min of specific dynamic movements. These specific movements depended on the test programmed to be performed first. Ballistic dynamic movements were performed before ROM measures, whereas prior to jump performance tests participants completed squats, lunges and vertical jumps in order to prepare the muscles to maximal performance. A 3-minute rest period followed the warm-up to avoid potential adverse effects on test performance.

#### Jumping performance tests

**Fig 1:**
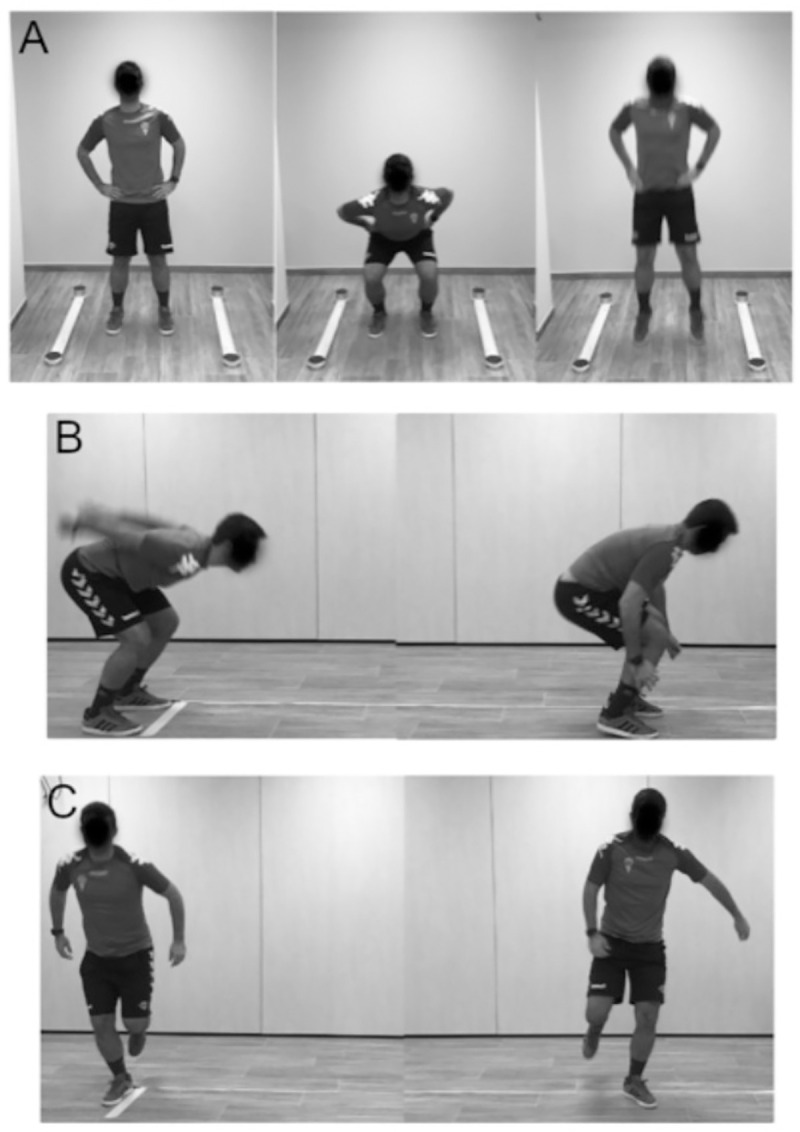
This figure depicts jumping tests performed in our study to assess bilateral countermovement jump [CMJ] (A), standing broad jump [SBJ] (B), and lateral jump [LJ] (C).

Jump testing comprised bilateral and unilateral countermovement jumps (CMJs) and standing broad jumps (SBJs). In addition, single leg lateral jumps (LJs) were registered (Fig 1). Participants performed two maximal trials for each jump, separated by a 45 s resting period [48]. For the CMJs, all participants were instructed to place their hands on their hips, which was followed by a vertical jump at maximal effort and landing in a vertical position, with their knees being flexed after landing [49]. A platform with infrared rays (Optojump Next, Microgate ®, Bolzano, Italy) was used [50] to measure jump height (cm) calculated as: h = gt/8 (h, height, cm; g, acceleration due to gravity, 9.81 m·s^-2^; t, flight time of the jump, s) [51]. For SBJs and LJs participants started from a standing position, swinging their arms and bending their knees to provide maximal forward frontal and lateral drive, respectively. Using a metric tape, jump-length was determined from the take-off line to the nearest point of landing contact (i.e. back of the heels) [52]. The highest height and distance registered were used for further statistical analysis.

#### Passive range of motion tests

**Fig 2:**
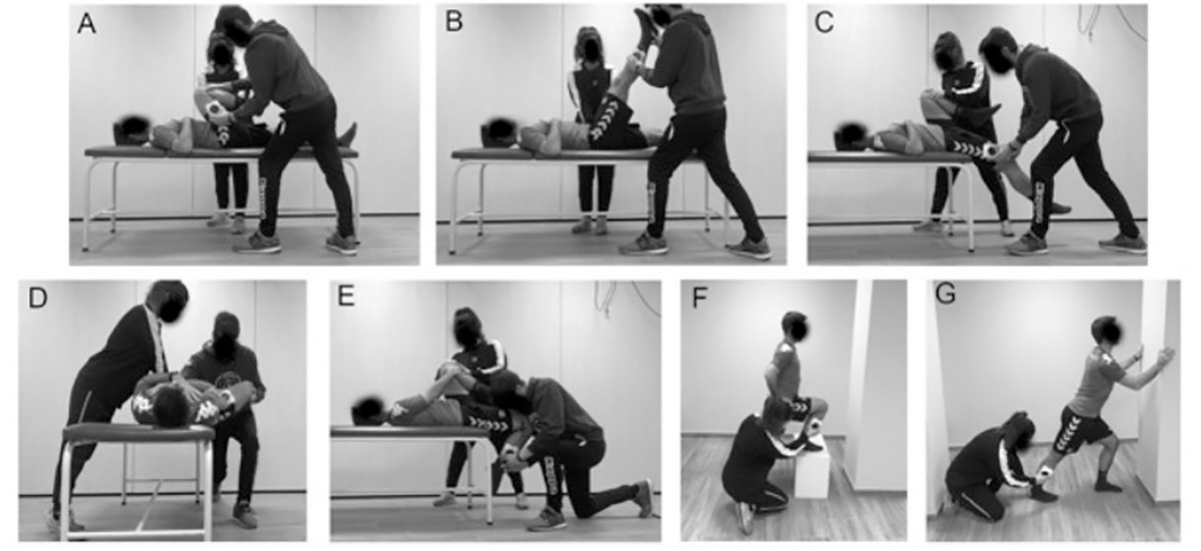
Lower limb passive range of motion testing. Positioning of the subjects performing passive ROM testing: (A) Passive hip flexion with flexed knee [HFFK]; (B) passive hip flexion with extended knee [HFEK]; (C) passive hip extension with flexed knee [HEFK]; (D) passive hip abduction with flexed knee [HAFK]; (E) passive knee flexion [KF]; (F) passive ankle dorsiflexion with flexed knee [ADFKF]; and (G) passive ankle dorsiflexion with extended knee [ADFKE].

A battery of passive ROM measures was performed on both the dominant and non-dominant legs, following methodology previously described [53]. This battery consisted of passive hip flexion with flexed (HFFK) and extended knee (HFEK); passive hip extension (HEFK) and abduction (HAFK) with flexed knee; passive knee flexion (KF) and ankle dorsiflexion with flexed (ADFKF) and extended knee (ADFKE) (Fig 2). These tests were selected because they have been considered appropriate by American Medical Organizations [54], and have been included in manuals of sports medicine and science based on anatomical knowledge, extensive clinical and sport experience, being its reliability previously demonstrated for team sport athletes [55] and healthy subjects [56–58]. For ROM measurements, a valid laser-guided digital goniometer (HALO medical devices, Ciudad, Australia) was used [59]. Mean score values from each test and limb were calculated based on records from two maximal trials for each ROM test and limb, and were used for further analysis. A 30 s rest interval was given between trials, limbs and test, and the goniometer was recalibrated before each trial. All tests were carried out under stable environmental conditions by the same two physical therapists, whose role was always the same. One or both of the following criteria determined the endpoint for each test: (a) palpable onset of pelvic rotation, and/or (b) the participant feeling a strong but tolerable stretch, slightly before the occurrence of pain. A 5-minute rest period was provided before starting jump testing.

### Statistical analyses

Test scores were initially recorded as means and standard deviations (SD). All data were checked for normality using the Shapiro–Wilk test, and reliability of all test measures was calculated using coefficient of variation (CV) ([SD/mean] x 100) [60]. For each unilateral test and participant, the limb showing higher jumping scores was defined as dominant, whereas the opposite limb was defined as non-dominant [61]. Jump asymmetries were calculated using a standard percentage difference equation for all tests: (dominant–non-dominant)/(dominant) × 100 [61]. ROM asymmetries were calculated as the ratio between legs with the following formula: (dominant / non-dominant). Dominant and non-dominant leg for ROM ratio calculation was determined based on test-specific (CMJ, SBJ) bilateral jump values. Parametric tests were performed when data were normally distributed, whereas equivalent non-parametric tests were used when data violated the assumption of normality. Student’s t-test for independent samples was performed in order to evaluate mean differences between sports (i.e. soccer and basketball) for jump performance and ROM parameters, and its asymmetry values; whereas Mann–Whitney U tests were performed when data were non-normally distributed. Practical significance was assessed by Cohen’s effect size (ES) calculated as d_s_ [62]. ES of above 0.8, between 0.8 and 0.5, between 0.5 and 0.2, and lower than 0.2 were considered large, moderate, small, and trivial, respectively. Pearson’s *r* correlations were conducted to establish the relationship between the inter-limb ROM ratio score and bilateral jump performance in CMJs and SBJs in basketball and soccer players when data were found to be normally distributed, whereas Spearman’s rho coefficients were calculated for non-normally distributed data. The significance level was set *p* < 0.05. All statistical tests were performed using the software package IBM SPSS Statistics, version 20.0 (IBM Corp., Armonk, N.Y., USA).

## Results

The statistical analysis for the comparison of jump performance and asymmetries between young soccer and basketball players did not reveal significant differences between sports, either for CMJs, or for SBJs and LJs (Table 1; *p* > 0.05 for all comparisons).

**Table 1:** Summary of results obtained during jumping tests.

		Soccer	Basketball	Mean differences (%)	*p*	ES
**CMJ (cm)**	Bi	30.92 ± 7.43 (24.02)	33.00 ± 6.79 (20.59)	6.73	0.249	0.29 small
	D	16.92 ± 4.01 (23.71)	18.41 ± 4.93 (26.75)	8.85	0.177	0.34 small
	ND	14.81 ± 3.97 (26.8)	16.21 ± 4.51 (27.80)	9.46	0.184	0.33 small
	Asymmetry	#12.56 ± 8.57 (68.21)	11.72 ± 8.22 (70.15)	-6.64	0.808	0.10 trivial
**SBJ (m)**	Bi	1.93 ± 0.23 (12.14)	2.03 ± 0.26 (12.99)	5.47	0.091	0.41 small
	D	1.67 ± 0.20 (12.11)	1.72 ± 0.26 (14.81)	3.38	0.317	0.22 small
	ND	1.57 ± 0.20 (13.06)	1.64 ± 0.24 (14.91)	4.40	0.215	0.32 small
	Asymmetry	#6.02 ± 4.91 (81.56)	#5.05 ± 3.82 (75.75)	-16.08	0.539	0.22 small
**LJ (m)**	D	1.51 ± 0.15 (10.03)	1.57 ± 0.20 (12.85)	3.74	0.221	0.35 small
	ND	1.43 ± 0.16 (11.19)	1.50 ± 0.19 (12.64)	4.51	0.138	0.41 small
	Asymmetry	#5.07 ± 4.05 (79.81)	4.30 ± 2.63 (61.11)	-15.19	0.645	0.22 small

Note.

#: not normal distribution; *p*: p-value; ES: effect size; Bi: bilateral; D: dominant; ND: non-dominant; CMJ: countermovement jump; SBJ: standing broad jump; LJ: lateral jump.

Values are shown as mean ± standard deviation (% coefficient of variation).

ROM values and comparisons between soccer and basketball players are shown in Table 2. Analysis of ROM data measured in lower limbs revealed significant differences between soccer and basketball players for HFFK test, both for the dominant (t_65_ = -3.16; *p* = 0.002) and non-dominant (t_65_ = -2.23; *p* = 0.029) legs. Young soccer players showed significantly lower HFFK ROM both for dominant (-4.35%) and non-dominant (-2.94%) legs. Significant differences between sports were also obtained for the HEFK test, both for the dominant (t_65_ = 3.12; *p* = 0.004) and non-dominant (t_65_ = 3.02; *p* = 0.001) legs. Young soccer players showed significantly greater HEFK ROM for dominant (24.89%) and non-dominant (24.57%) legs. Moreover, significant differences were observed between young soccer and basketball players for the ratio of HAFK (t_65_ = -2.25; *p* = 0.028). Whereas soccer players showed higher ROM HEFK values in the dominant leg (ratio = 1.02 ± 0.08), basketball players showed higher values in the non-dominant leg (ratio = 0.97 ± 0.11). No significant differences were observed between young soccer and basketball players for the remaining ROM measures (Table 2, *p* > 0.05 for all comparisons).

**Table 2:** ROM values comparisons between soccer and basketball players.

		Soccer	Basketball	Mean difference (%)	*p*	ES
**HFFK**	D	142.43 ± 7.74 (5.44)	148.63 ± 8.10 (5.45)	4.35	0.002**	0.79 moderate
** **	ND	144.38 ± 8.36 (5.79)	148.63 ± 6.45 (4.34)	2.94	0.029*	0.56 moderate
	Ratio	0.99 ± 0.04 (4.17)	1.00 ± 0.05 (4.81)	1.01	0.189	0.23 small
**HFEK**	D	^#^78.76 ± 11.73 (14.9)	^#^81.41 ± 11.76 (14.45)	3.36	0.381	0.23 small
	ND	79.81 ± 10.92 (13.69)	80.70 ± 9.61 (11.91)	1.12	0.732	0.09 trivial
** **	Ratio	0.99 ± 0.07 (7.17)	1.01 ± 0.09 (9.29)	2.02	0.26	0.25 small
**HEFK**	D	^#^13.26 ± 4.71 (35.5)	9.96 ± 3.42 (34.38)	-24.89	0.004**	0.78 moderate
** **	ND	^#^12.86 ± 4.55 (35.34)	^#^9.70 ± 3.62 (37.36)	-24.57	0.001**	0.75 moderate
	Ratio	1.07 ± 0.31 (28.65)	1.06 ± 0.32 (30.15)^#^	-0.93	0.573	0.03 trivial
**HAFK**	D	75.69 ± 7.89 (10.43)	75.63 ± 10.89 (14.40)	-0.08	0.98	0.01 trivial
	ND	74.24 ± 7.08 (9.54)	78.22 ± 9.87 (12.62)	5.36	0.059	0.48 small
** **	Ratio	1.02 ± 0.08 (7.73)	0.97 ± 0.11 (11.01)	-4.90	0.028*	0.54 moderate
**KF**	D	112.24 ± 11.64 (10.37)	112.48 ± 12.84 (11.42)	0.21	0.936	0.02 trivial
** **	ND	112.99 ± 13.31 (11.78)	110.81 ± 15.13 (13.66)	-1.93	0.537	0.15 trivial
	Ratio	1.00 ± 0.07 (7.45)	1.02 ± 0.09 (8.72)	2.00	0.244	0.25 small
**AFEK**	D	36.29 ± 4.64 (12.77)	35.59 ± 6.25 (17.55)	-1.93	0.624	0.13 trivial
	ND	35.44 ± 4.57 (12.9)	34.63 ± 7.60 (21.95)	-2.29	0.623	0.14 trivial
** **	Ratio	1.03 ± 0.11 (10.49)	^#^1.05 ± 0.15 (14.23)	1.94	0.98	0.16 trivial
**AFFK**	D	36.59 ± 5.70 (15.58)	38.11 ± 7.21 (18.93)	4.15	0.339	0.24 small
** **	ND	34.8 ± 5.31 (15.25)	37.37 ± 6.87 (18.39)	7.39	0.089	0.43 small
** **	Ratio	1.06 ± 0.14 (13.07)	1.02 ± 0.08 (8.20)	-3.77	0.175	0.33 small

Note

#: not normal distribution; *p*: p-value; ES: effect size; D: dominant; ND: non-dominant; HFFK: hip flexion with flexed knee; HFEK: hip flexion with extended knee; HEFK: hip extension with flexed knee; HAFK: hip abduction with flexed knee; KF: knee flexion; AFEK: ankle flexion with extended knee; AFFK: ankle flexion with flexed knee

*: significant results (p < 0.05)

**: significant results (p < 0.01).

Values are shown as mean ± standard deviation (% coefficient of variation).

No significant associations were observed between bilateral CMJ and SBJ performance, and the ratio of ROM observed at lower limb joints (Table 3; *p* > 0.05 for all associations tested).

**Table 3:** Results obtained for the associations between ratio of ROM (ROM_D/ROM_ND) and bilateral jump performance for CMJ and SBJ. respectively.

	Bilateral CMJ		Bilateral SBJ	
	Soccer	Basketball	Soccer	Basketball
**HFFK**	r = 0.024; *p* = 0.882	r = 0.045; *p* = 0.825	r = 0.232; *p* = 0.149	rho = -0.035; *p* = 0.864
**HFEK**	r = -0.152; *p* = 0.349	r = 0.353; *p* = 0.071	r = 0.165; *p* = 0.307	r = 0.364; *p* = 0.062
**HEFK**	r = 0.106; *p* = 0.517	rho = -0.157; *p* = 0.435	r = -0.049; *p* = 0.762	r = -0.065; *p* = 0.748
**HAFK**	r = -0.305; *p* = 0.056	r = 0.184; *p* = 0.358	r = -0.206; *p* = 0.202	r = -0.032; *p* = 0.875
**KF**	r = 0.017; *p* = 0.919	r = 0.127; *p* = 0.527	r = -0.037; *p* = 0.822	r = -0.128; *p* = 0.523
**AFEK**	r = -0.092; *p* = 0.572	rho = -0.358; *p* = 0.067	r = 0.046; *p* = 0.779	rho = 0.229; *p* = 0.251
**AFFK**	r = -0.190; *p* = 0.241	r = -0.016; *p* = 0.937	r = -0.129; *p* = 0.429	r = 0.148; *p* = 0.461

Note: rho: Spearman’s correlation coefficient; *p*: p-value; HFFK: hip flexion with flexed knee; HFEK: hip flexion with extended knee; HEFK: hip extension with flexed knee; HAFK: hip abduction with flexed knee; KF: knee flexion; AFEK: ankle flexion with extended knee; AFFK: ankle flexion with flexed knee.

## Discussion

The aim of the present study was threefold: i) to compare bilateral and unilateral jumping capacity between youth soccer and basketball players, along with inter-limb jumping asymmetries; ii) to compare passive ROM of lower limbs between youth soccer and basketball players, and ROM ratio between dominant and non-dominant leg; and iii) to explore the associations between bilateral jump performance and ROM ratio for each sport modality. Significant differences between youth soccer and basketball players were observed for specific passive ROM measured at hips. However, no significant differences between sports were obtained for jumping performance, and the association analysis revealed no significant correlations between ROM ratio and bilateral jump performance, either for soccer, or for basketball youth players.

Jumping capacity has been established as one of the most prevalent tests in soccer and basketball due to its inherent role as a principal action of the game, and as an important neuromuscular power output measure [63, 64]. Since soccer and basketball players are required to win duels in the air to gain advantage over the opponent, there is a need to optimize jumping ability from early ages, attending to specific sport demands [65, 66], thus it is a training goal for coaches aiming at improving both strength and sport-specific technical skills [13]. In line with this, vertical jump height is crucial for an efficient ball heading (i.e., when an athlete attempts to play the ball in the air with his or her head) in soccer players, with interception, a head pass, clearance or shot the final purpose of the action [67, 68], whereas horizontal jump distance has been positively correlated with successful rebounds and block actions per game in basketball players [69]. The results of the present study revealed no significant differences between soccer and basketball youth players for bilateral and unilateral multiplanar jumping performance. Our results are in line with the lack of significant differences for CMJ height previously reported by Kollias, Panoutsakopoulos and Papaiakovou [16] when comparing soccer (30.1 ± 2.7 cm) and basketball (30.0 ± 4.5 cm) adult male players. Moreover, CMJ jump values observed in this study are in line with those reported by Rodríguez-Rosell et al. [70] for soccer (U15: 32.0 ± 4.8 cm; U18: 36.7 ± 4.6 cm) and basketball players (U15: 31.9 ± 4.9 cm; U18: 33.0 ± 5.9 cm) from similar age categories. However, to the best of our knowledge, this study is the first to compare SBJ and LJ performance between young soccer and basketball players, finding no differences between sport-modalities. According to the obtained results, similar training models for the improvement of jumping ability (e.g. power and plyometric training-based exercises) could have been adopted in both sport disciplines in an effort to achieve athletes’ highest performance levels [13, 70–72]. Finally, with regard to multidirectional jump inter-limb asymmetries, a lack of significant differences between young soccer and basketball players was observed. These results might be explained by the common asymmetrical movement patterns that can be observed in both sport-modalities, which are determined by similar limb function and limb dominance related to each sport-specific demands [73, 74]. Altogether, these results suggest that there is no need to develop sport-specific training programs focused on reducing inter-limb asymmetries, although it would be reasonable to develop an individualized approach, aimed at preventing inter-limb differences for players showing asymmetry values associated with greater injury risk [75].

Screening tests and exercises aimed at improving flexibility are a crucial part of the injury prevention strategies used by coaches in team sports, based on their capacity to achieve and test for optimal ROM values, respectively [76]. The applicability of these injury prevention strategies has been explored in soccer [76–78] and basketball [79]. However, there is still controversy regarding the relationship between flexibility and both injury prevention and sport performance [80, 81]. After performing a battery of seven ROM tests for lower extremities, the results obtained in the current study revealed significant differences between soccer and basketball players only for hip joint ROM measures. Soccer players showed significantly higher HEFK ROM values for the dominant (mean difference: 24.89%) and non-dominant (mean difference: 24.57%) leg. These results, then, suggest that young soccer players present greater flexibility for hip flexor muscles, when compared to young basketball players. It is reasonable to suggest these differences are due to the different physical and technical demands of each sport and consequent specific flexibility training outcomes. For instance, soccer players are required to have higher levels of flexibility from hip flexors during the backswing phase of the instep kicking action, since an optimal level of stretch from the antagonist muscle (i.e. hip flexor) combined with an optimal contraction of the agonist muscle (i.e. hip extensor) allows higher displacement, which affects kicking outcomes achieved at high ball speed [82]. Furthermore, improved ROM hip extension has been associated with increased kicking speed in young male soccer players, while reduced extensibility of tissues around this joint is thought to be related to injury risk [29, 83]. Indeed, some authors have also suggested that hip flexor flexibility could be a significant independent predictor of hamstring injury related to sprinting mechanics, leading to the proposal of maximal hip flexion as a protective mechanism [30, 84].

Our results revealed smaller HFFK ROM both for the dominant (mean difference: 4.35%) and non-dominant (mean difference: 2.94%) leg for soccer players compared to young basketball players, suggesting lower levels of gluteus flexibility for the former. Interestingly, since gluteus muscle is prone to weakness, leading to adverse changes in movement kinematics, increased risk of injury, and decreased sport performance [85–87], exercises aimed at improving gluteus muscle activation are in the top five developed in soccer’s injury prevention programs [77]. In addition, hip extensor strengthening has been recommended as a hamstring injury prevention strategy [88], since hamstring muscles are exposed to injury risk when high levels of force, velocity and power production are needed to produce maximal sprint acceleration [89]. Thus, recent research has suggested hip extensors act as a determinant compensatory muscle for horizontal force production, allowing performance to be maintained and the protection of hamstring muscles from injury in fatigue condition [88, 90]. Thus, considering that soccer players are more exposed to the training and repetition of sagittal-specific soccer skills (i.e. maximal power sprint) whereas basketball players perform more movements in the frontal plane (i.e. changes of direction) [6], it is reasonable to suggest that hip extensors are more strengthened in soccer players as a result of adaptation to this continuous contraction of hip extensor muscles, thus leading to diminished hip extensor flexibility [34, 40]. However, due to the limited scientific evidence focused on the assessment of passive ROM and flexibility status in team sports, it is difficult to discuss the obtained results. Thus, future research is needed in order to gather more evidence regarding potential passive ROM differences among team sports athletes, and their effect on specific sport performance and injury prevention.

As for the values of inter-limb passive ROM ratio (dominant vs non-dominant leg), between-sport branches significant differences were only observed for the HAFK test. Whereas young soccer players showed greater hip passive abduction for the dominant leg, their young basketball counterparts showed greater hip passive abduction for the non-dominant leg. Based on the independent between-sport branches comparison performed for the dominant and non-dominant HAFK values, it is clear inter-limb passive ROM HAFK ratio differences resulted from sport-specific values observed for the non-dominant leg. Whereas similar HAFK values were observed for the dominant leg between soccer and basketball young players (mean difference = -0.08%; *p* = 0.98), nearly significant differences were observed for the non-dominant leg (mean difference = 5.36%; *p* = 0.059). Although no clear explanation has been found in the literature for this observation, and thus further research is needed, it is plausible that differences observed for the HAFK ratio might result from sport-specific development of non-dominant HAFK, due to training and match play demands associated with each sport-modality. In this regard, In this regard, Lopez-Valenciano et al. [40] reported inter-limb passive hip abduction differences > 6° in favour of the dominant limb in outfield soccer players, suggesting it resulted from adaptations to repetitive performance of soccer-specific actions (i.e., ball kicking, and control). Whereas soccer requires players to perform a number of repeated unilateral actions (e.g. ball kicking), preferentially executed with the dominant leg [32], basketball players must perform actions in multiple directions and situations [91]. However, neither young soccer players, nor young basketball players showed inter-limb asymmetries on ROM values larger than the cut-off value (10%) proposed as indicative of injury risk [61, 92–94], which would suggest there is no need for sport-specific injury prevention routines, although an individual approach to the problem might be worth taking, as previously suggested by Bishop et al. [75] with practitioners designing individualized training interventions when needed.

Assessing inter-limb asymmetry has become one of the main lines of research among sport scientists aiming at improving athletes’ performance [95]. For this purpose, strength- and jump-tests are usually applied to compare between-limbs function or performance, and to examine its effects on performance of team sport specific actions (i.e. changes of direction or sprint) [19, 96]. While recent literature has reflected that evidence based on jumping asymmetries are inconclusive [97], strength and ROM asymmetries seem to negatively affect performance and sport-specific skills [21, 52, 98], being further associated with injury risk in team sport athletes [29, 99]. Whereas strength measures are harder to be implemented by coaches, since they require more complex and expensive equipment, ROM methods could be a suitable alternative for the assessment of inter-limb imbalances. However, to the best of our knowledge no previous research has associated inter-limb asymmetries based on passive ROM measurements with jumping performance. The results obtained in the present study show a lack of associations between passive ROM ratio measured in the dominant and non-dominant legs, and bilateral jump performance, either for the CMJs, or for the SBJs. These evidences might result from the lower limb performance functional asymmetry shown by soccer and basketball players as a result of the sport-specific asymmetrical physical requirements, and the variable nature of asymmetry reported for young players from both sports [75]. Given that passive ROM asymmetries have no detrimental impact on jumping performance, it could be concluded that reducing flexibility asymmetry scores is not a relevant strategy to improve jumping in this specific population. Nevertheless, further research should analyze the relationship between ROM asymmetry and sprint and change of direction performance in order to establish whether reducing ROM asymmetry scores allows performance to be improved in this specific young athletes’population.

The main limitation of this study was the small sample size with regard to the number of participants from each sport modality, and the between-group variability resulting from the specific team characteristics in soccer and basketball. Furthermore, future research should test whether results observed in this study are replicated for each age category, controlling for the training tasks performed by each team, aiming at evaluating whether potential between-sport task similarities could explain the lack of jumping differences observed in this research. However, this is the first study comparing lower limb passive ROM between young soccer and basketball players, and thus it establishes normative values for future research. On the other hand, although only associations between inter-limb asymmetries based on passive ROM measure and jump performance have been analyzed in this study, relevant results have been obtained suggesting coaching staff to test whether potential relationships exist between passive ROM asymmetries and other determinant sport-specific actions in team sports.

## Conclusions

Young soccer and basketball male players present no significant differences in multiplanar bilateral and unilateral jumping performance and jumping asymmetries, likely due to the multicomponent nature of both sport modalities and the implementation of common training strategies. However, adaptions resulting from repetitive performance of sport-specific actions (i.e. ball kicking) lead to passive ROM differences between these young populations, although there is no need for the implementation of training strategies specifically aimed at increasing ROM values, since inter-limb ROM asymmetries are smaller than thresholds suggested as risk for injury (10–15% difference). In addition, the lack of association between ROM asymmetries and bilateral jump performance suggests training programs focused on minimizing inter-limb ROM differences would not lead to an improvement of multidirectional jump performance.

These findings suggest that staffs of both sport disciplines could include similar training models for the improvement of jumping ability (i.e., power and plyometric training-based exercises) in an effort to achieve athletes’ highest performance levels. Additionally, reducing inter-limb ROM asymmetries does not seem to be a key strategy to increase multidirectional jump performance. Finally, coaches should develop an individualized approach to each player aiming to reduce inter-limb asymmetries and consequently, the risk of injury.

## Supporting information

S1 Data (XLSX)
